# Pilonidal sinus of the cheek: an extremely rare clinical entity—case report and brief review of the literature

**DOI:** 10.1186/s13256-020-02561-z

**Published:** 2021-02-10

**Authors:** B. N. Adhikari, S. Khatiwada, A. Bhattarai

**Affiliations:** 1 Plastic and Reconstructive Surgery Division, Department of Surgical Oncology, B P Koirala Memorial Cancer Hospital, Bharatpur 7, Nepal; 2 Department of Anatomy, Chitwan Medical College, Bharatpur 10, Nepal; 3 Chitwan Medical College, Bharatpur 10, Nepal

**Keywords:** Case report, Pilonidal sinus, Cheek sinus, Rare location, Unusual site, Extracoccygeal pilonidal disease

## Abstract

**Background:**

Pilonidal sinus is commonly seen at the sacrococcygeal region and few other sites, usually located at the midline, at areas where hairs collect or near protuberances or some adjacent rubbing surfaces. Its presence elsewhere is uncommon. We share an interesting case of a recurrent discharging sinus from the cheek bulge of a male which turned out to be a pilonidal sinus containing tuft of hairs on exploration and wide excision.

**Case presentation:**

A 37 years old hirsute male presents to us with a non-healing discharging sinus at the bulge of the cheek. Exploration after a course of antibiotics showed 2 subcutaneous cavities with tuft of hairs. The area was excised along with a margin, thorough irrigation and curettage was done and the wound was closed primarily; a Z-plasty was incorporated in the central part to break the resultant suture line. Histopathological examination was done to confirm the diagnosis and rule out an off-midline dermoid cyst or an underlying/coexisting malignancy. Post-operative course was uneventful. The patient has been recurrence free for 1.5 years and is satisfied with the nature of the scar.

**Conclusions:**

Pilonidal sinus of the cheek bulge is an extremely rare entity. Complete excision and clinical suspicion are important for cure of this nagging ailment, especially at unexpected areas.

## Background

Pilonidal sinus (PNS) refers to a subcutaneous sinus which contains hair. It is an acquired condition caused by penetration of hair fragments into the skin. This hair containing cavity usually is asymptomatic and the only sign of its presence may be a small pit on the surface of the skin [1, 2]. Due to infection of its content, it may present clinically either as a recurrent discharging sinus or an abscess.

PNS is a relatively common condition mostly seen in hirsute males at the sacrococcygeal region in approximately 26/100,000 population [2]. PNS of the face are very rare and are usually reported in the literature as case reports or small series of primary as well as recurrent cases. Over the face, the nose is most commonly affected followed by other areas like the chin, mandible, buccal area, forehead, ear, eyelid and the cheek [1]. Only two cases of PNS over the cheek, in the preauricular area, have been reported in the literature till date [2, 3].

We present a case of a recurrent discharging sinus of the cheek which turned out to be a pilonidal sinus pathologically and not a dermoid, tubercular or a malignant cyst.

## Case presentation

On February 2019, a 36 year old hirsute male presented to us with a recurrent (several episodes since the last 2 years) discharging sinus over the right cheek which was operated twice previously. He did not have tooth ache or pain while moving the upper and the lower jaws. There was no significant medical or psychosocial history and his family support was considered satisfactory. There was no history of Tuberculosis (TB); similarly history of insect bite or pricks over the area was lacking. On examination, the right cheek contained a fairly good amount of normal looking hair and showed some signs of inflammation at the preauricular area around a small opening. The discharge from the opening was turbid, non-bloody, slightly foul smelling and did not contain any sebum, hair or yellowish granules. A provisional diagnosis of infected dermoid cyst was made and the patient discharged home with anti-inflammatory drugs and a 7 days oral course of Flucloxacillin and Metronidazole after radiologically ruling out Temperomandibular joint (TMJ), gums, teeth, parotid or maxilla/mandible as a potential underlying source of infection. The patient was planned for excisional biopsy with 2 mm margin after a week of local infection control (Table 1).

**Table 1:** Timeline of relevant events

1st noticed (painful bulge)	January 2017
Discharge noted	February, 2017
Operated 1st (?Incision and drainage)	May, 2017
Operation 2nd	December, 2017
Self-Expression of pus from wound	March, 2018
Operation 3rd	April–May, 2018
Operation 4th (Our center)	February, 2019
Regular follow up—wound healed	June, 2019
Patient traced—telephone interview (recurrence—yes/no; response—no)	March, 2019
Telephone follow-up (recurrence—Yes/No; response—No), satisfaction with final outcome and scar (satisfied, not satisfied—would/would not like to undergo a minor procedure to improve the scar; Response—Satisfied)	August, 2020

**Fig. 1:**
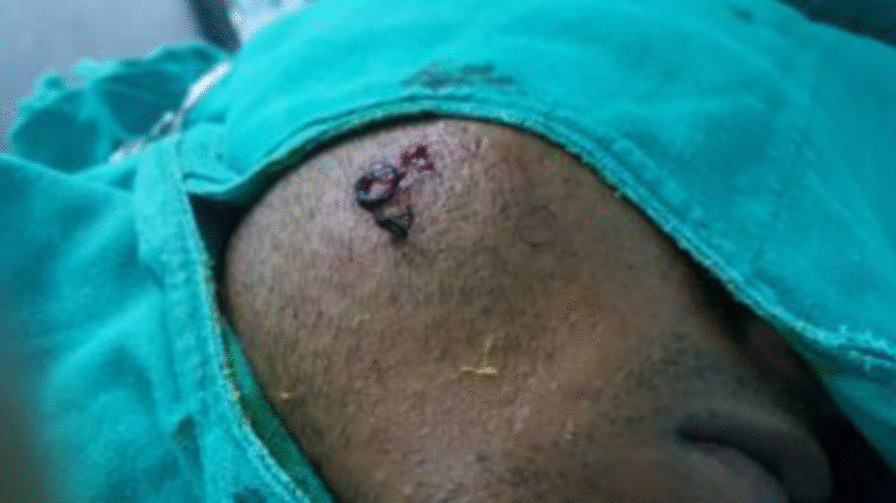
Pilonidal sinus of the cheek

After a week, the wound was explored under local anesthesia. We found 2 (3 × 2 cm and 1 × 1 cm) interconnected sinus cavities containing 2 rolls of hair and 2 tracks leading to the skin (Fig. 1).

Pictures were taken and the patient was sedated and rest of the operation was completed under IV anesthesia. The resultant defect, after wide excision of the sinuses, was converted to a lenticular shaped defect along the Langer's line and the wound was closed in two layers after curettage and a thorough wash. To avoid a long curvilinear scar, a small Z plasty was planned at the center. The patient was discharged in the evening and called daily for the first 3 days to check for signs of inflammation and local collection.

Outcome and follow-up—The postoperative course was, fortunately, uneventful. The sutures were removed on 7th day of discharge. Histopathological confirmation of pilonidal sinus was collected at 3 weeks and further follow-up visits were scheduled monthly. The patient was lost to follow-up after 4 months. After 1.5 years, the patient was traced and telephonic interview was done to inquire about any signs suggestive of a recurrence. The patient was also asked about satisfaction with the result of the surgery as well as the nature and satisfaction about the facial scar. The patient had signed an informed consent for use of the data and photos made during and after surgery.

## Discussion

Pilonidal disease is described back as far as 1833, when Mayo described a hair-containing cyst located just below the coccyx. Hodge coined the term "pilonidal" from its Latin origins in 1880 and today, pilonidal disease describes a spectrum of clinical presentations, ranging from asymptomatic hair-containing cysts and sinuses to large symptomatic abscesses that have some tendency to recur [4].

The etiology of pilonidal sinus remains unclear. There are two main theories regarding its etiology—acquired and congenital. In general, at least three conditions need to be fulfilled for a pilonidal sinus to occur: first is hair in the skin and, second, some kind of wrinkled skin, such as the natal cleft or a scar. The third condition is a mixture of hormonal and hygienic problem [4, 5].

Pilonidal sinus is typically encountered in the sacrococcygeal region, but rare cases have been described at other unusual sites in around 2.2% cases [6], notably the axilla [7, 8], suprapubic area [9], periumbilical zone [10–13], inguinal region [8, 11], neck [8, 11], periungal region [14], clitoris [15], penis [8, 16–18], nipple [11, 19, 20], intermammary region [11, 12], interdigital space of foot and hand [2, 21–24], scalp [8, 11], scrotum [25], endoanal canal [26, 27] and the face. The first reported case of extrasacrococcygeal pilonidal sinus (ESPS) was located in the interdigital region and reported in 1942 [6]. The nose, chin, mandible, buccal area, forehead and the cheek are the reported areas of ESPS in the face. Only two cases of PNS over the cheek, in the preauricular area, have been reported in the literature till date. The 1st reported case was of a 22 years old male from Iraq in 2016 [3] and the 2nd case was of a 36 years old male from Romania in 2017 [2]; both have had these problematic sinuses over the cheek for a long time and got cured with the final excision.

Doll et al. [5] argues against attributing trauma as playing the pivotal role in all cases of PNS; O’Sullivan earlier believed otherwise and described a facial PNS, located over the mandible, where trauma seemed to play a major role in the inception [28]. Nevertheless, for PNS of the cheek to occur, repeated mechanical trauma from shaving coupled with contributions from high testosterone level, repeated folliculitis and intrusion of hair fragment through the damaged overlying skin seems to play the major contributory role [6, 9].

Majority of facial PNSs occur in males. Males have facial hair which needs repeated shaving—this leads to abundance of rootless razor sharp short hairs which are implicated in the pathogenesis of facial PNS [1, 29]. PNS sometimes occurs in drivers and hair dressers as the former are exposed to prolonged sitting and standing cycles while the latter deals with hair frequently. There is no reported job which increases the risk for developing facial PNS [1].

An ingrown hair inciting repeated irritation of the skin thereby causing its intrusion was excluded as a differential diagnosis because of the presence of many hairs deep inside the excised cyst. Moreover, there were no black lines and patient refused the history of habitual hair pulling [30]. Usually, an ingrown hair is also more common in people who have very curly or coarse hair, which appears because the hairs have curled around and grown back into the skin, instead of rising up from it [2].

Skin tumors like Basal Cell Carcinoma (BCC) may mimic Pilonidal Sinus [31], or more frequently, these chronically infected cysts may predispose the individual to develop different cutaneous malignancies (notably, Squamous Cell Carcinoma, BCC, sweat gland adenocarcinoma and verrucous carcinoma) over the area [25]. Due to occasional and unsuspected presentation of Tuberculosis (in our part of the world) and malignancy, any chronic non-healing wound should prompt an astute clinician to rule out these commoner entities with a tissue diagnosis. We opted for a wide local excision of the suspected area with 2 mm margin for confirmation.

In the present case, there was a surgical history of repeated drainage of a cheek swelling about 3 months back which might be the cause of inadvertent entry of hairs in the wound. This cyst may have been misdiagnosed as an abscess and operated twice, which was not the definitive surgery. So the patient continued having discharge from the operative site and was finally diagnosed as pilonidal sinus after the last operation. The patient is satisfied with both the scar as well as the outcome of the surgery.

The standard method of management of facial PNS is excision and primary closure which is usually performed under general or local anesthesia. This may differentiate facial PNS from PNS of other areas where many non-surgical modalities exists. The skin of the face is more lax allowing direct closure which may also eliminate a crease or groove. Leaving a wound over the cheek for secondary closure or having an underlying dead space results not only in a cosmetically inferior scar but also increases the chances of a recurrence. Adequately excised PNS of the face usually do not recur [1]; however, rare cases of recurrence of the cyst have been reported in the scalp [32], interdigital space [33], penis [33], axilla [11], sternum and inguinal region [11].

## Limitations of the study

No preoperative pictures were available as the sinus was initially considered trivial and operation planned under local anesthesia as last case of the day (fear of infection and contaminating other cases). Patient was not physically available for late follow up and quantification of scar and satisfaction outcome was not done.

## Conclusions

Although PNS is very rare in the cheek bulge, it should be included in the differential diagnosis of subcutaneous nodule and chronic sinus when hair is seen inside the cyst cavity or when there is history of some surgical intervention. Wide excision of the sinus tract with a margin and primary or a delayed primary closure is recommended for a better cosmetic outcome.

## Data Availability

Yes.

## Abbreviations

BCC: Basal cell carcinoma
ESPS: Extrasacrococcygeal pilonidal sinus
PNS: Pilonidal sinus
TB: Tuberculosis
TMJ: Temperomandibular joint
